# Correcting for photodestruction in super-resolution optical fluctuation imaging

**DOI:** 10.1038/s41598-017-09666-4

**Published:** 2017-09-05

**Authors:** Yves Peeters, Wim Vandenberg, Sam Duwé, Arno Bouwens, Tomáš Lukeš, Cyril Ruckebusch, Theo Lasser, Peter Dedecker

**Affiliations:** 1 Department of Chemistry, KU Leuven, Leuven, Belgium; 2 Laboratoire d’Optique Biomédicale, STI – IBI, École Polytechnique Fédérale de Lausanne, Lausanne, Switzerland; 3 Department of Radioelectronics, Faculty of Electrical Engineering, Czech Technical University, Prague, Czech Republic; 4 LASIR CNRS UMR, Université de Lille, Lille, France

## Abstract

Super-resolution optical fluctuation imaging overcomes the diffraction limit by analyzing fluctuations in the fluorophore emission. A key assumption of the imaging is that the fluorophores are independent, though this is invalidated in the presence of photodestruction. In this work, we evaluate the effect of photodestruction on SOFI imaging using theoretical considerations and computer simulations. We find that photodestruction gives rise to an additional signal that does not present an easily interpretable view of the sample structure. This additional signal is strong and the resulting images typically exhibit less noise. Accordingly, these images may be mis-interpreted as being more visually pleasing or more informative. To address this uncertainty, we develop a procedure that can robustly estimate to what extent any particular experiment is affected by photodestruction. We also develop a detailed assessment methodology and use it to evaluate the performance of several correction algorithms. We identify two approaches that can correct for the presence of even strong photodestruction, one of which can be implemented directly in the SOFI calculation software.

## Introduction

The diffraction limit in far-field optical imaging, first described by Ernst Abbe^1^, is no longer an insurmountable barrier to the spatial resolution of fluorescence microscopy. During the last decades, several revolutionary advances have been made to observe structures with increased spatial resolution^2–5^. Techniques were developed based on either repeated single-molecule localization (e.g. PALM^6, 7^, STORM^8^) or patterned illumination (e.g. SIM^9^, ISM^10^) possibly in combination with saturation effects (e.g. STED^11, 12^, RESOLFT^13, 14^, non-linear SIM^15^).

Another addition to the super-resolution imaging field is super-resolution optical fluctuation imaging (SOFI)^16^. SOFI relies on reversible and independent fluctuations in fluorescence emission, or “blinking”, known to occur in virtually all fluorophores. These fluctuations are recorded through the rapid acquisition of multiple fluorescence images from the same region of the sample. Due to the presence of blinking, the active set of emitters continuously varies over the duration of the experiment. Each of the fluorescence images therefore presents complementary information on the sample structure. SOFI extracts this extra information by calculating the cumulant of the fluorescence intensities observed in each detector pixel, resulting in a super-resolved image. The exact resolution improvement depends on the order of the cumulant calculation: calculating the *n*th-order cumulant results in an image with a $\sqrt{n}$ or *n*-fold resolution improvement in three dimensions, depending on whether additional processing is performed^17^. In addition to the improved spatial resolution, SOFI also reduces emission not originating from the labels (background signal) and improves contrast^16, 18^. Compared to other super-resolution techniques, SOFI does not require special imaging equipment, requires minimal sample preparation steps^19, 20^, and can work well under a wide range of imaging conditions, such as a low signal-to-noise ratio (SNR)^21^ or the presence of steady state diffusion^22^. Another advantage is that the entire image formation process is described by a simple analytical model^16, 23, 24^. As a trade-off, the resolution improvement in SOFI is often limited to a factor 2 to 5 depending on the label used. This arises because at heart SOFI relies on statistical estimation to extract the super-resolved image. This estimation process becomes increasingly reliable when more frames of experimental data are included. Since higher-order calculations are inherently more noisy, more data-frames are required to produce an image with a comparable SNR. Since any dataset is necessarily of finite duration, the highest order of analysis that still results in interpretable images will dictate the achievable resolution^25^.

An important limitation is that the blinking of the fluorophores must be resolvable. Given the typical temporal resolution of tens of milliseconds available in many camera-based imaging systems, this means that only blinking on these time scales or slower can be used for SOFI. In practice, SOFI has been found to work well for dyes ranging from quantum dots^16^ and organic dyes^23, 26^ to fluorescent proteins^18, 27, 28^. Recently, it was also shown that SOFI can be used to read out a new class of fluorescent biosensors^29, 30^, resulting in the first approach that can visualize reversible signalling activity in live cells with a spatial resolution better than the diffraction limit^30^.

From a theoretical viewpoint, SOFI delivers reliable images as long as the following conditions are met: (1) the fluorophores display two optically distinguishable states (typically one fluorescent and the other non-emitting); (2) the fluorophores switch between these states repeatedly and independently; and (3) the signal is stationary, in other words, there is no long-term trend in the fluorescence emission. While these assumptions are reasonable, a key problem is the fact that all known fluorophores undergo photodestruction or photobleaching, meaning that their fluorescence signal is irreversibly lost at some point in time. Although the degree of photodestruction can be limited to some extent by adjusting the experimental set-up, e.g. using timed irradiation sequences that allow the relaxation of non-fluorescent states^31, 32^, it is never fully eliminated. This invalidates the second and third assumptions of SOFI imaging, since the fluorophores are no longer independent, and neither is the signal stationary. This violation of the fundamental requirements of SOFI imaging clearly casts doubt on the usefulness or reliability of the technique in actual use.

In this work, we investigate the impact of photodestruction on the accuracy and reliability of SOFI imaging. We find that photodestruction gives rise to a strong signal in the SOFI image. Because the signal is strong, it leads to images that have a higher signal-to-noise ratio (SNR) and that may appear more informative or more pleasing to the eye. However, this photodestruction-induced signal does not present an easily-interpretable view on the sample structure, effectively distorting the image. Given this important insight, we develop a procedure that can robustly estimate the extent of photodestruction signal in an arbitrary SOFI experiment. We also set out to identify strategies that can correct for photodestruction, which we evaluate quantitatively. We identify two approaches that work well across a broad range of conditions, one of which can be readily implemented in SOFI software, where its functioning is transparent to the end user.

## Results

### Photodestruction and the SOFI imaging model

For completeness we briefly review the theory of SOFI imaging. At heart, the pixel values in any SOFI image are cumulants of the intensity distribution observed in detector pixels.

As we know from ref. 16, the fluorescence signal *F* at position *r* and time *t* and its associated fluctuation *δF* (*r*, *t*) are given by

1 $$F\,(r,t)=\sum _{k=1}^{N}\,U(r-{r}_{k})\cdot {\varepsilon }_{k}\cdot {s}_{k}\,(t)$$

2 $$\delta F\,(r,t)=F\,(r,t)-{\langle F(r,t)\rangle }_{t}$$

Where N is the number of emitters, *U*(*r*) is the point spread function (PSF) of the microscope, *ε*_*k*_ is the molecular brightness, 〈…〉_*t*_ denotes averaging over all times *t* and *s*_*k*_(*t*) a switching function that is equal to one if the *k*-th fluorophore is fluorescent at time *t*, and zero if the molecule is in a non-fluorescent state.

The simplest approach to SOFI imaging calculates the intensity cumulants for each detector pixel separately. For example, using Eqs 1 and 2 we can write the second-order auto-cumulant as

3 $$\begin{array}{rcl}A{C}_{2}(r,\tau ) & = & {\langle \delta F(r,t+\tau )\cdot \delta F(r,t)\rangle }_{t}\end{array}$$

4 $$\begin{array}{ccc} & = & \sum _{k=1}^{N}\,\sum _{j=1}^{N}\,U(r-{r}_{k})\,U(r-{r}_{j})\cdot {\varepsilon }_{k}\cdot {\varepsilon }_{j}\cdot \langle\delta {s}_{k}\,(t)\,\delta {s}_{j}\,(t+\tau ){\rangle}_{t}\end{array}$$

5 $$\begin{array}{rcl} & = & \sum _{k=1}^{N}\,{U}^{2}(r-{r}_{k})\cdot {\varepsilon }_{k}^{2}\cdot {\langle \delta {s}_{k}(t)\delta {s}_{k}(t+\tau )\rangle }_{t}\end{array}$$

The composition of Eq. 5 is exemplary of the behavior of any SOFI calculation. In general any cumulant used in SOFI imaging will consist of an additive contribution for each fluorophore, but with a PSF that is sharper than the PSF of the wide-field system (respectively *U* ^2^(*r*) and *U*(*r*) in this example). The additional terms in the cumulant (*ε*_*k*_ and 〈*δs*_*k*_(*t*)*δs*_*k*_(*t* + *τ*)〉_*t*_ in this case) merely act as weighting factors which we assume are the same for all fluorophores with identical photophysical behavior. The overall SOFI signal is therefore linear with respect to the emitter density.

However, Eq. 5 is valid if and only if 〈*δs*_*k*_(*t*)*δs*_*l*_(*t* + *τ*)〉_*t*_ = 0 for *k* ≠ *l*, in other words, when the dynamics of the fluorophores are independent. Fundamentally, the presence of photodestruction invalidates this assumption. Since all emitters undergoing photodestruction transfer to a permanent dark state during the experiment, they become de-facto correlated. This means that Eq. 5, and its fundamental importance to the interpretation of SOFI images, no longer holds.

We can make this more systematic using a simple thought experiment: suppose that we are observing a sample with non-blinking fluorophores that undergo photodestruction. To an approximation, the signal in pixel A can then be described as

6 $${s}_{A}\,(t)=A\,\exp \,[-\tfrac{t}{{\tau }_{{\rm{bl}}}}]$$

where *τ*_bl_ is the characteristic time constant of the photodestruction process. If we now plug this expression into Eq. 3, and assume that the measurement is performed over a duration *T*, then the SOFI signal is given by

7 $$A{C}_{2}\,(\tau )={A}^{2}\,\mathop{\underbrace{\frac{{\tau }_{{\rm{bl}}}{e}^{-\tfrac{2T+\tau }{{\tau }_{{\rm{bl}}}}}\,({e}^{\tfrac{T}{{\tau }_{{\rm{bl}}}}}-1)\,(T+{e}^{\tfrac{T}{{\tau }_{{\rm{bl}}}}}\,(T-2{\tau }_{{\rm{bl}}})+2{\tau }_{{\rm{bl}}})}{2{T}^{2}}}}\limits_{{\rm{constant}}\,{\rm{for}}\,{\rm{all}}\,{\rm{detector}}\,{\rm{pixels}}}$$

Equation 7 signifies that photodestruction contributes a SOFI signal, but that the image that comes out of this calculation is equivalent to simply squaring a diffraction limited image. Clearly this photodestruction signal does not provide any super-resolution information, and only serves to contaminate the informative SOFI signal that originates from the reversible fluorophore dynamics. In practice, a somewhat more involved calculation strategy is used to produce SOFI images^17, 33, 34^, though these conclusions remain valid.

### Quantifying the extent of photodestruction signal in experimental data

**Figure 1:**
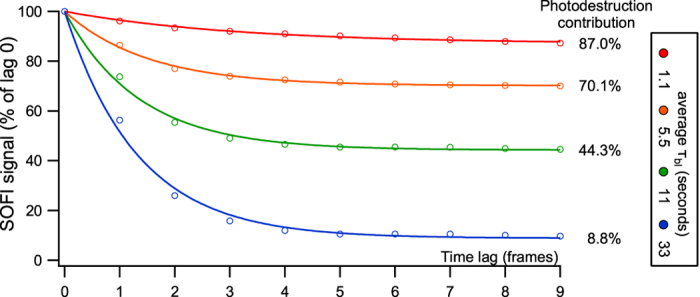
Estimation of photodestruction contribution to a measured SOFI signal. Datasets with different photodestruction rates were simulated and the SOFI signal was calculated for increasing lag times *τ*.

The SOFI experimentalist may wonder to what extent the signal in a particular experiment has been contaminated by photodestruction. Fortunately, it is possible to determine this in a way that can be applied to nearly any particular measurement. The crucial insight is that the SOFI cumulants can be calculated for different time lags *τ* (Eq. 3). For short time lags (small *τ*), the SOFI value will contain both signals arising through fluorophore blinking and through photodestruction. However, the SOFI signal for longer time lags (larger *τ*) will contain only the contribution of photodestruction, assuming that the blinking dynamics are fast compared to the timescale of photodestruction. This assumption is reasonable and likely to be valid given the requirement for reversible blinking. As Fig. 1 shows, an increased propensity to photodestruction translates to larger cumulant amplitudes for longer lag times. The amplitude of the plateau for large *τ* relative to the cumulant value for *τ* = 0 allows the direct assessment of how much of the signal is contaminated due to photodestruction.

### Quantifying the effect of photodestruction using simulated data

**Figure 2:**
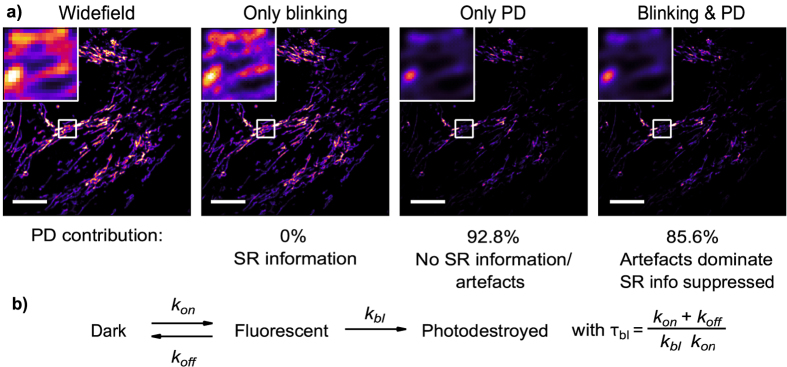
(**a**) Visualisation of the contribution of blinking and/or photodestruction to SOFI. All images are derived from simulated data sets of 500 frames based on HeLa cells with labelled DAKAP (mitochondria). The widefield image is the average image of this sequence while the remaining images are second order SOFI images. Photodestruction (PD) was simulated with *τ*_*bl*_ = 1.1 s. SR: super-resolution. Scale bar 10 *μ*m. Color map: extended black body. (**b**) Photophysical scheme.

We turned to simulated datasets with known ground-truth sample structure to rigorously quantify the effect of photodestruction. We started by simulating fluorescence images based on an actual SOFI image showing mitochondria, in the presence and absence of photodestruction. The simulated fluorophores obeyed the photophysical scheme shown in Fig. 2b (experimental procedure detailed in the methods section). In this photophysical model, *k*_bl_ is a microscopic rate constant for bleaching, while the observed overall fluorescence intensity decays as exp(−*t*/*τ*_bl_). Figure 2 summarizes the key findings, which match with the theoretical considerations above:

1. Photodestruction gives rise to a strong SOFI signal that does not correspond to the actual structure of the sample.
2. The additional SOFI signal through photodestruction results in images that are less noisy. These images may thus be selected as ‘more pleasing’ or ‘more informative’.
3. In the extreme case, a SOFI image can be observed even in the case where the fluorophores do not blink, but only bleach. However, this image does not present easily-interpretable information on the sample structure. As we discuss further below, this arises because photodestruction destroys the linear dependence of the SOFI signal on the emitter density.

**Figure 3:**
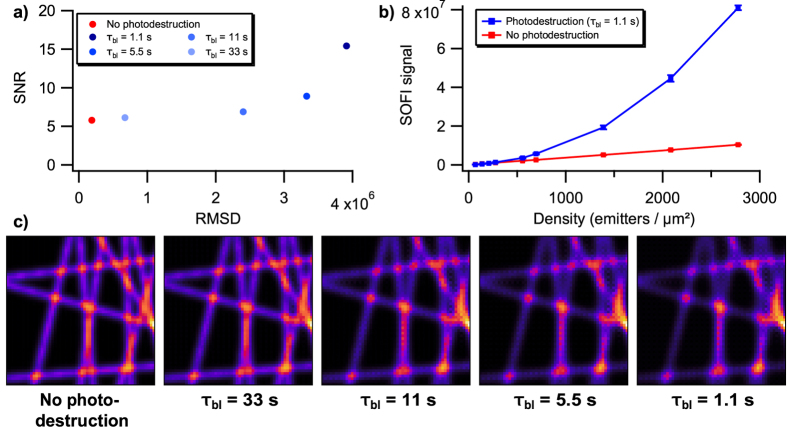
Effect of photodestruction on 2^*nd*^ order SOFI calculations. (**a**) 2D plot of SNR and RMDS values of data with varying degrees of photodestruction. Photodestruction was simulated by assigning the emitters with a characteristic survival time (*τ*_*bl*_). Datasets consisted of 500 frames (**b**) Effect of photodestruction on 2^*nd*^ order SOFI signal from different label densities. (**c**) Average 2^*nd*^ order SOFI images with varying degrees of photodestruction. Colour map: extended black body.

We then turned our attention to a more systematic evaluation, performing many simulations of artificial line structures (Fig. 3). Our previous result (Fig. 2) had clearly highlighted the need to evaluate both the accuracy (the correctness of the structure) and the precision of the imaging (the SNR). Accordingly, we quantified the accuracy by calculating the root mean square deviation (RMSD) between each SOFI image and the correct sample structure, where the correct structure was given by a SOFI image calculated on a dataset without photodestruction and with a large number of simulated fluorescence images. RMSD is an appropriate metric for this comparison because we correct for any scaling factors, and did not observe any absolute offsets in the SOFI signals. We quantified the precision by repeating each simulation 100 times and calculating the signal-to-noise ratio associated with the images, following the procedure described in ref. 34.

Figure 3a quantitatively confirms that photodestruction increases SNR as well as RMSD. This proves that the signal caused by photodestruction does not faithfully represent the structure (RMSD) and is strong compared to the signal caused by blinking (SNR). Crucially, the images with the highest distortion (highest RMSD) also have the highest SNR. This increase in RMSD mainly arises because the SOFI signal no longer depends linearly on the emitter density (Fig. 3b), as can also be seen by visual inspection of Fig. 3c. Performing an analogous procedure for 3^*rd*^ order SOFI imaging leads to very similar observations, except that the effect of photodestruction is even more profound (Supplementary Fig. S1).

### Correcting for photodestruction

#### Possible strategies

We next turned our attention to the identification of strategies that can correct the effect of photodestruction. Similar concerns have been encountered in other techniques that make use of the statistical analysis of data that is assumed to be stationary, such as fluorescence correlation spectroscopy, potentially allowing us to adapt these solutions to SOFI.

One strategy that has been proposed is the use of *batching* ^24, 26, 35^. In this approach, the input image sequence is divided into a number of smaller image sequences, also known as sub-sequences or batches, that are analysed independently. This results in multiple SOFI images for every dataset, that are then averaged to yield a single SOFI image. Typically the sizes of these batches are set to some default value, e.g. grouping 25, 50, or 100 fluorescence images into a single batch. The effect of photodestruction will be strongly reduced if the duration of these sub-sequences is longer than the blinking correlation time, but shorter than the photodestruction time.

Ries *et al*.^36^ have proposed a correction for photodestruction in the context of fluorescence correlation spectroscopy. The core idea is to transform the acquired data such that the mean and variance of the fluorescence intensities are constant in time. The first step is to fit the observed intensity trace, *F*(*t*_*i*_), with a model function *f*(*t*_*i*_). Typically *f*(*t*_*i*_) is taken to be a single- or bi-exponential function. The corrected intensity trace *F* ^*c*^(*t*_*i*_) is then obtained using:

8 $${F}^{c}({t}_{i})=\frac{F({t}_{i})}{\sqrt{f({t}_{i})/f\mathrm{(0)}}}+f\mathrm{(0)}\,(1-\sqrt{f({t}_{i})/f\mathrm{(0)}})$$

We will refer to this correction as the *moment*-*preserving correction*, since it strives to preserve the first and second moment of the intensity distribution.

Another possibility is to correct the pixel-intensity values by dividing the uncorrected value by the value extracted from a normalized fit of the pixel-intensity trace^37^ (further referred to as *multiplication correction*):

9 $${F}^{c}({t}_{i})=F({t}_{i})\frac{f\mathrm{(0)}}{f({t}_{i})}$$

In addition, we will also investigate a correction by simple subtraction. The idea here being that the intensity trace is assumed to be composed of two parts: the fluctuations of the emitters superimposed on the exponential decay caused by photodestruction. By modelling and subtracting this decay we attempt to eliminate its effects^38^.

10 $${F}^{c}({t}_{i})=F({t}_{i})-f({t}_{i})$$

#### Evaluation of the photodestruction corrections

**Figure 4:**
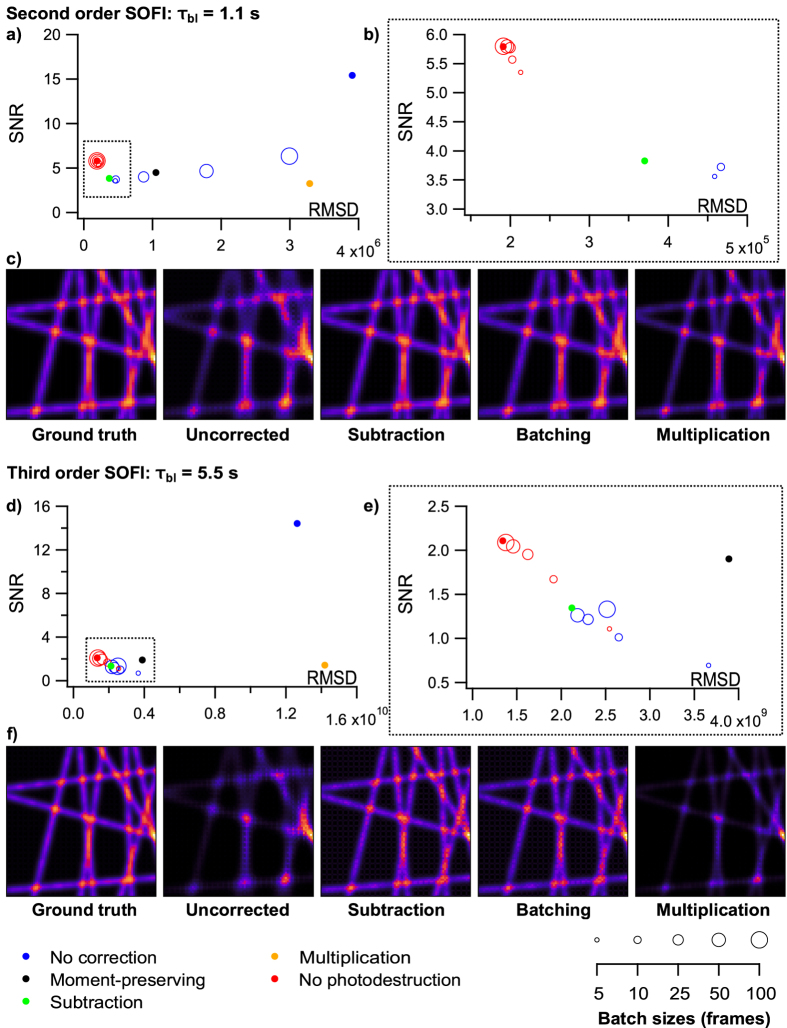
Evaluation of different photodestruction-correcting methods on simulated data. (**a**) 2^*nd*^ order SOFI with fluorophores assigned a *τ*_*bl*_ value of 1.1 s. Batch sizes of 100, 50, 25, 10, and 5 frames were examined and are visualized by different marker sizes. (**b**) Close-up of the region in dotted box in (**a**). (**c**) Images of certain datasets to serve as a visual reference for RMSD values. (**d**–**f**) correspond to (**a**–**c**) respectively for 3^*rd*^ order simulations with a *τ*_*bl*_ value of 5.5 s. All images are averaged SOFI images of 100 repetitions. A batch size of 25 was used to generate the batching corrected image.

Figure 4a–c and Supplementary Figs S2 and S3 analyse the performance of the different correction methods on second order SOFI imaging, using different photodestruction rates. A similar analysis is made for third order imaging in Fig. 4d–f and Supplementary Figs S5 and S6.

Overall, we find that the performance of the proposed methods varies dramatically. The subtraction method achieves the best results, with high SNR and low RMSD values. The batching correction also performs well, though the optimal size of the batches depends on the rate of photodestruction. The batching procedure has the advantage of being model-free, and achieves a performance very close to that of the subtraction method. We find that use of the batching approach with batch sizes of 25 to 50 frames results in SOFI images with excellent SNR and RMSD values. This conclusion holds for both second and third order SOFI imaging. The optimal values of the batch sizes can be found by fitting the experimental intensity traces with an exponential function to determine *τ*_bl_, and making reference to Fig. 4 and Supplementary Figs S2, S3, S5 and S6.

When analysing the SOFI signal from different label densities for the various correction methods, the subtraction method is the best at restoring the linear correlation between SOFI signal and label density (Supplementary Fig. S4). It also causes the least distortions when applied to data without bleaching (both lines overlap in Supplementary Fig. S4). A close second is the moment-preserving method, while the multiplication correction is very ineffective. Batching is also capable of retrieving this linear dependency, particularly when using batch sizes of 25 frames or smaller.

### Experimental data

**Figure 5:**
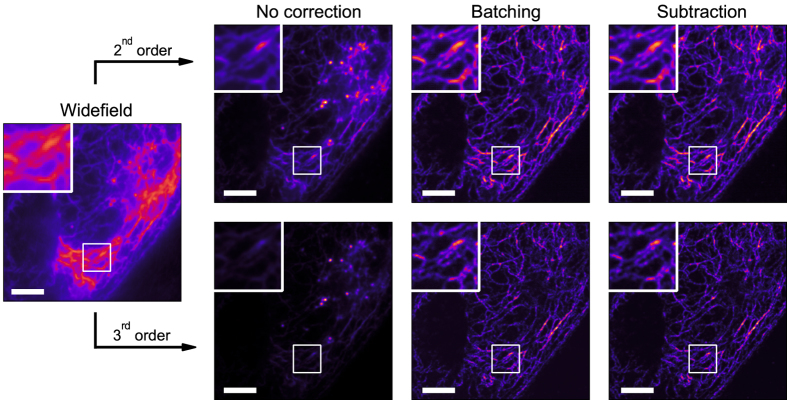
Comparison of correction methods on 2^*nd*^ and 3^*rd*^ order SOFI images of experimental data. An image sequence of 4,000 frames of HeLa cells expressing Dronpa-Krt19 was recorded. The widefield image is an average image of this sequence. A batch size of 25 frames was used to generate the batching-corrected SOFI image. Scale bar 5 *μ*m.

We applied the best performing correction methods to an experimental dataset where Dronpa was fused to keratin-19 (krt19) and expressed in HeLa cells (as detailed in the methods section). Both 2^*nd*^ and 3^*rd*^ order (Fig. 5) SOFI images were calculated based on corrected and uncorrected data. In both cases, uncorrected data yields a low quality SOFI image where certain structures are no longer visible due to the overwhelming contribution of photodestruction (74.8% for second order and 80.6% for third order calculations) to the SOFI signal. When correcting for this effect, we retrieve this information and clearly increase the quality of the SOFI images, especially with 3^*rd*^ order calculations. The photodestruction contribution is effectively eliminated. At these photodestruction rates (*τ*_*bl*_ approximately 9 s), all correction approaches perform similarly, with little differences between SOFI images of differently corrected data, similar to the results of simulated data.

## Conclusion

We have examined the effect of photodestruction on the accuracy and precision of SOFI imaging. We find that photodestruction introduces correlations between the fluorophores that violate the key assumptions of SOFI. These additional correlations give rise to a strong SOFI signal, which gives rise to images that can appear more visually pleasing due to a lower noise content. However, this bleaching signal does not provide any super-resolved information and cannot be easily interpreted. However, by making use of the different timescales of blinking and photodestruction, we were able to develop a straightforward procedure that can estimate the contribution of photodestruction to any experimental SOFI image.

We then developed a framework that allowed us to evaluate both the precision and accuracy of simulated SOFI images influenced by arbitrary photodestruction parameters. Using this framework, we proposed and assessed a variety of correction methods. We find that the subtraction method proposed here performed very well, closely followed by the batching method. Because batching presents an easy to use and simple solution that requires no additional calculations or manipulations of the data, but greatly increases SOFI image quality (see e.g. Fig. 5), we recommend its general use as a necessary and sufficient correction for photodestruction. While the batch size depends on the specifics of the photodestruction and blinking kinetics, our results suggest that a batch size of 25 to 50 frames will work under the majority of experimental conditions. We have now updated our Localizer software package to make use of batching with a default batch size of 50 frames.

## Methods

### Simulations

**Figure 6:**
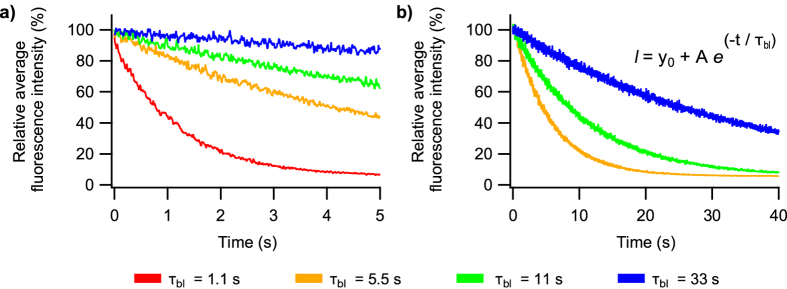
Intensity profile of single simulations with different photodestruction rates for (**a**) 2^*nd*^ and (**b**) 3^*rd*^ order SOFI calculations. A total of 500 and 4,000 images with 10 ms intervals were constructed for the 2^*nd*^ and 3^*rd*^ order calculations respectively. The relative average intensity of the image is plotted in function of the image number.

To study the differences between the various correction approaches, we simulated fluorescence images where the fluorophores were located on 10 randomly oriented and intersecting lines, both with and without photodestruction of the emitters. All simulated data sets were based on a 32 × 32 pixel detector, with an optical pixel size of 100 nm. 20,000 emitters were simulated with an on-time ratio of 9%, meaning that each fluorophore spent approximately 9% of its pre-photodestruction time in the fluorescent state in which they can emit 30 photons/ms. Each simulated fluorophore was assumed to be independent of all the other fluorophores, with fluorescence dynamics that follow the scheme shown in Fig. 2b and that are modelled as a continuous-time Markov chain with an average blinking rate of *k* = 1/(*τ*_on_ + *τ*_off_), with *τ*_on_ = 10 ms and *τ*_off_ = 100 ms. The camera exposure time was set at 10 ms. The PSF is simulated to be 2D Gaussian with its standard deviation determined by the numerical aperature (1.4) and the wavelength (520 nm). Shot noise, camera offset (1000), and EM gain multiplication (50) and noise were included. A background 10 photons per pixel (Poisson distributed). Photodestruction was simulated by defining the characteristic time constant of the bleaching process, (parameter *τ*_*bl*_ in Fig. 2b), which is the expected time before photodestruction occurs. Photodestruction was assumed to be distributed mono-exponentially. Survival times (*τ*_bl_; 1.1, 5.5, 11, and 33 s) were arbitrarily chosen to evaluate the performance of the correction methods at a range of photodestruction rates. As a reference value, the experimental data shown in Fig. 5 displayed a *τ*_bl_ of about 9 s. Typical simulated intensity traces are visualized in Fig. 6. From each simulated dataset, we derived corrected datasets by applying the described correction methods. We then calculated SOFI images for each of these using the Localizer software package^39^. To evaluate the batching method we examined batch sizes of 100, 50, 25, 10, and 5 frames.

Different label densities were simulated in a single image by dividing a 32 × 32 pixel detector in nine 10 × 10 squares with label densities ranging from 70 to 2800 emitters/*μm* ^2^. After SOFI calculations, the average SOFI signal of a 6 × 6 pixel square at the centre of each 10 × 10 square was calculated to exclude edge effects. Simulations were repeated 10-fold. A *τ*_*bl*_ value of 1.1 s was used for simulations with photodestruction.

DAKAP labelled HeLa cells were simulated with emitter positions based on a SOFI image from experimental data. 750,000 emitters were simulated on a 512 × 512 pixel detector. Photodestruction was simulated with *τ*_*bl*_ = 1.1 s. For the data set with only photodestruction, *k*_*off*_ was set to zero, eliminating blinking. All other settings were identical to those described above.

### SNR and RMSD determination

All simulations were repeated a 100-fold and averaged to determine the SNR and RMSD, measures for precision and accuracy respectively. We determine the SNR as described elsewhere^34^. RMSD allows us to quantitatively compare different images to a reference image, which was the average SOFI image of 100 simulations of large image sequences (5,000 images for 2^*nd*^ order and 20,000 images for 3^*rd*^ order) without photodestruction. In addition, data sets without photodestruction but with the same amount of fluorescence images as data with photodestruction (500 images for 2^*nd*^ order and 4,000 images for 3^*rd*^ order) were also simulated, allowing us to identify the best possible SNR and RMSD when confined to these sequence sizes. Prior to RMSD calculation, scale factors were corrected by equalizing the image averages. We used Igor Pro (WaveMetrics, Inc., Lake Oswego, OR, USA) and the Localizer software package^39^ to run all simulations, calculations and data analysis.

### Experimental quantification of photodestruction

To evaluate the amount of photodestruction in experimental data the following procedure is followed. First an experimental dataset is loaded into the freely available Localizer software^39^. At this time, for second order SOFI 10 distinct SOFI images are produced from the data using a time lag varying from 0 to 9 in the calculation. For higher order SOFI calculation, which can contain multiple different time lags, a similar procedure is followed where all time lags are set to 0 except for one, which is then varied between 0 and 9. Next, the mean value of each SOFI image is plotted against the time-lag, normalized to 100% for the image at lag 0. The curve that results can be fitted reasonably well to a mono-exponential decay for a wide array of experimental conditions (Eq. 11) using a least-squares fitting procedure. Though on occasion values for the contribution of photodestruction to the signal where found to be slightly negative (without physical meaning) after correction for photodestruction at which point the contribution of blinking was arbitrarily set to 0%. The resulting fit directly reports on the percentage of signal caused by photobleaching (% bleaching) as well as a constant which relates to the photochemical behavior of the dye (*τ*_blink_).

11 $${\rm{S}}{\rm{O}}{\rm{F}}{\rm{I}}\,{\rm{s}}{\rm{i}}{\rm{g}}{\rm{n}}{\rm{a}}{\rm{l}}=100-{\rm{ \% }}\,{\rm{b}}{\rm{l}}{\rm{e}}{\rm{a}}{\rm{c}}{\rm{h}}{\rm{i}}{\rm{n}}{\rm{g}}\,(1-\exp \,(-\frac{{\rm{T}}{\rm{i}}{\rm{m}}{\rm{e}}\,{\rm{l}}{\rm{a}}{\rm{g}}}{{\tau }_{{\rm{b}}{\rm{l}}{\rm{i}}{\rm{n}}{\rm{k}}}})$$

The curves in Fig. 1 where obtained by performing simulations of 500 frames as described earlier, with the photodestruction rate of the simulation mentioned in the figure.

### Experimental data

The plasmid with Dronpa fused to keratin19 (pBE-Dronpa-Krt19) was derived from plasmid pQE-rsCherryRev1.4-Krt19 (kindly provided by Jakobs S.). First BamHI and EcoRI restriction sites were inserted for easy cloning. Using standard cloning techniques, rsCherryRev1.4 was replaced with Dronpa. HeLa cells were cultured as described elsewhere^27, 28^ and transfected with pBE-Dronpa-Krt19 using FuGene 6 according to the manufacturers instructions. Imaging of live HeLa cells occurred approximately 24 hours after transfection. Prior to imaging, samples were washed three times using 2 mL PBS and stored in 2 mL HBSS. We used a commercial Cell TIRF microscope (Olympus) as described in refs 27 and 28. Power of a 488 nm laser was set at 8% (80% setting + ND1 filter) in quasi-TIRF configuration. Exposure time was 10 ms (to achieve this, the camera chip was cropped to 256 × 256 pixels) and EM gain was 600. In total, 4,000 images were recorded. No 405 nm light was used.

### Data availability statement

The datasets generated and/or analysed during the current study are available from the corresponding author on reasonable request.

## Electronic supplementary material

Supplementary information
