# Supplementary material for: Correcting for photodestruction in super-resolution optical fluctuation imaging
Source: Sci Rep. 2017 Sep 5;7:10470. doi: 10.1038/s41598-017-09666-4 (PMC5585228; doi:10.1038/s41598-017-09666-4)
Supplement: Supplementary file 1 — Supplementary information [file 41598_2017_9666_MOESM1_ESM.pdf]

Correcting for photodestruction in super-resolution optical fluctuation imaging - Supplementary information

Yves Peeters, Wim Vandenberg, Sam Duwé, Arno Bouwens, Tomaš Lukeš, Cyril Ruckebusch, Theo Lasser, and Peter Dedecker

Contents

1 Effect of uncorrected photodestruction on third order SOFI imaging **2**

2 Correcting photodestruction in second order SOFI simulations **2**

3 Correcting photodestruction in third order SOFI simulations **5**

1 Effect of uncorrected photodestruction on third order SOFI imaging

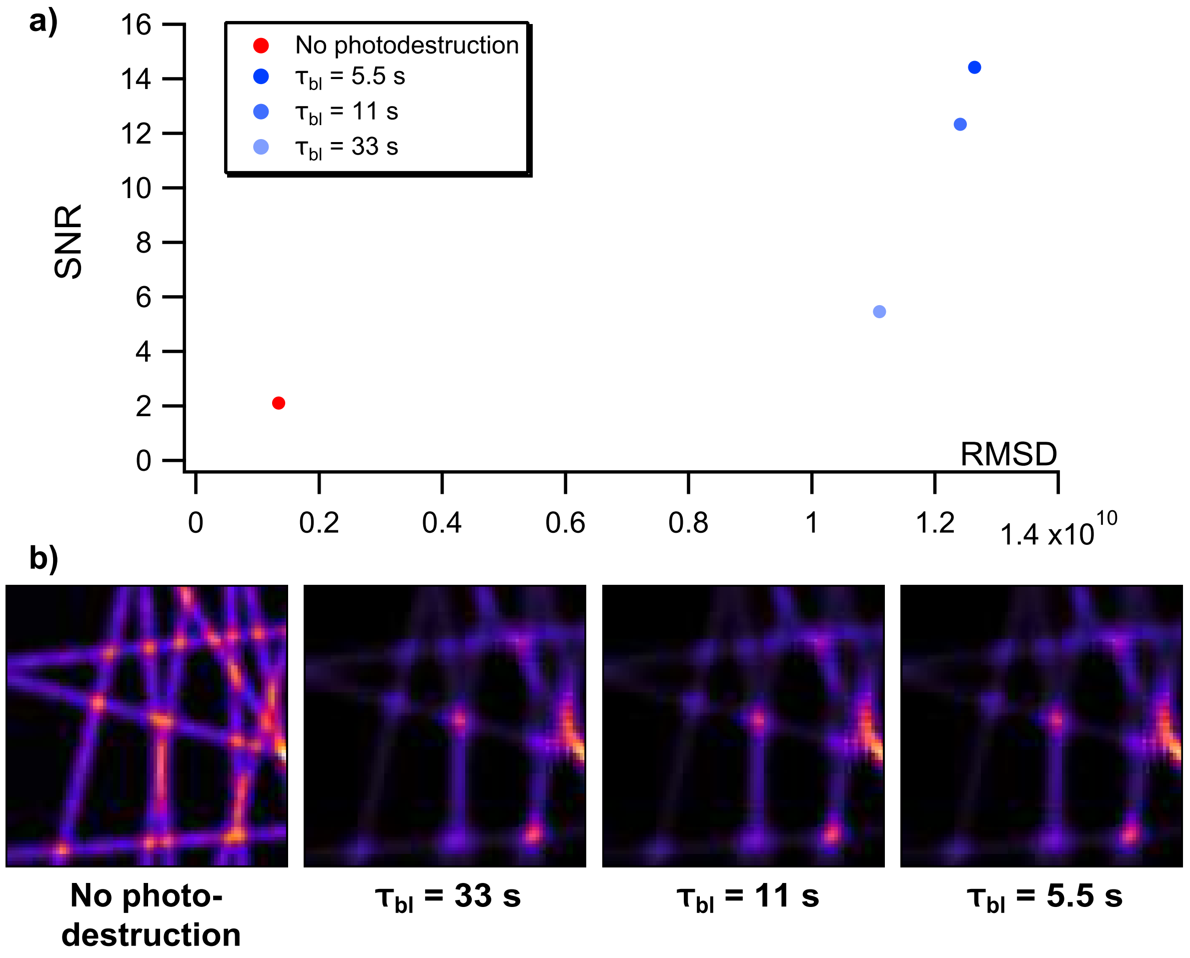

Figure S1: Effect of photodestruction on 3nd order SOFI calculations. (a) 2D plot of SNR and RMDS values of data with varying degrees of photodestruction. Photodestruction was simulated by defining a characteristic time constant of the photodestruction (τ_{bl}). The average SOFI image of 100 simulations of 20,000 frames without photodestruction was used as a reference to determine RMSD values. (b-e) Average 3nd order SOFI images with varying degrees of photodestruction (lower τ_{bl} values correspond to more photodestruction). SOFI images of 100 simulations were averaged to yield these images.

2 Correcting photodestruction in second order SOFI simulations

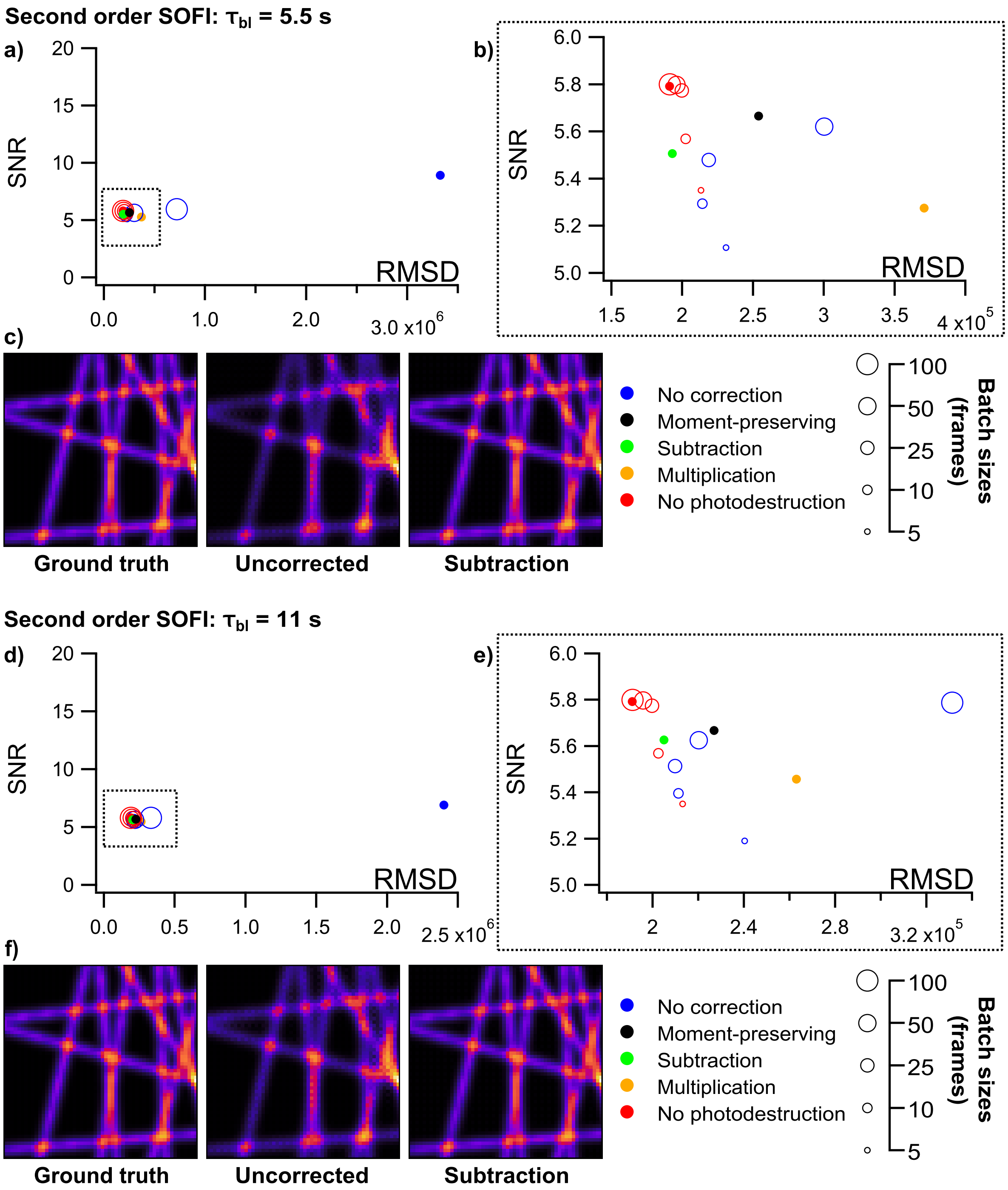

Figure S2: (a) Evaluation of different photodestruction-correcting methods on simulated data with fluorophores assigned a τ_{bl} value of 5.5 s for 2nd order SOFI calculations. (b) Close-up of region in dotted box in (a). (c) Images of certain datasets to serve as a visual reference for RMSD values. These images are averaged SOFI images of 100 repetitions. (d-e-f) correspond to (a-b-c) respectively for simulations with a τ_{bl} value of 11 s. Batch sizes of 100, 50, 25, 10, and 5 frames were examined and are visualized by different marker sizes.

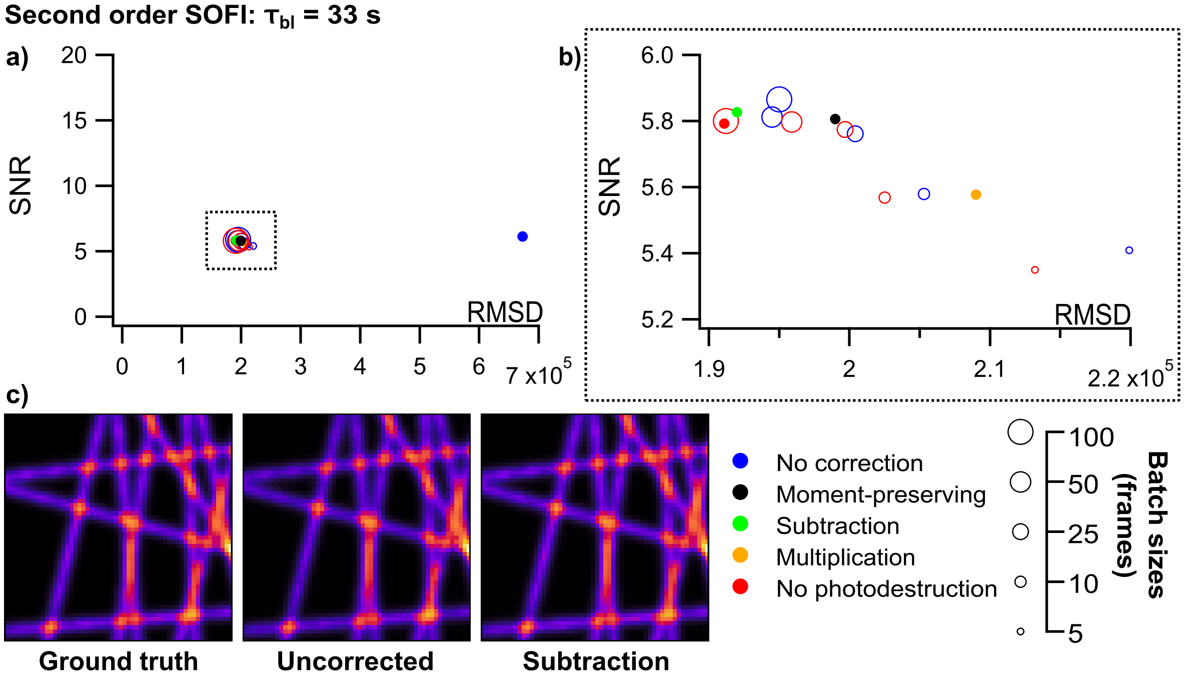

Figure S3: (a) Evaluation of different photodestruction-correcting methods on simulated data with fluorophores assigned a τ_{bl} value of 33 s for 2nd order SOFI calculations. Batch sizes of 100, 50, 25, 10, and 5 frames were examined and are visualized by different marker sizes. (b) Close-up of region in dotted box in (a). (c) Images are shown for certain datasets to serve as a visual reference for RMSD values. These images are averaged SOFI images of 100 repetitions.

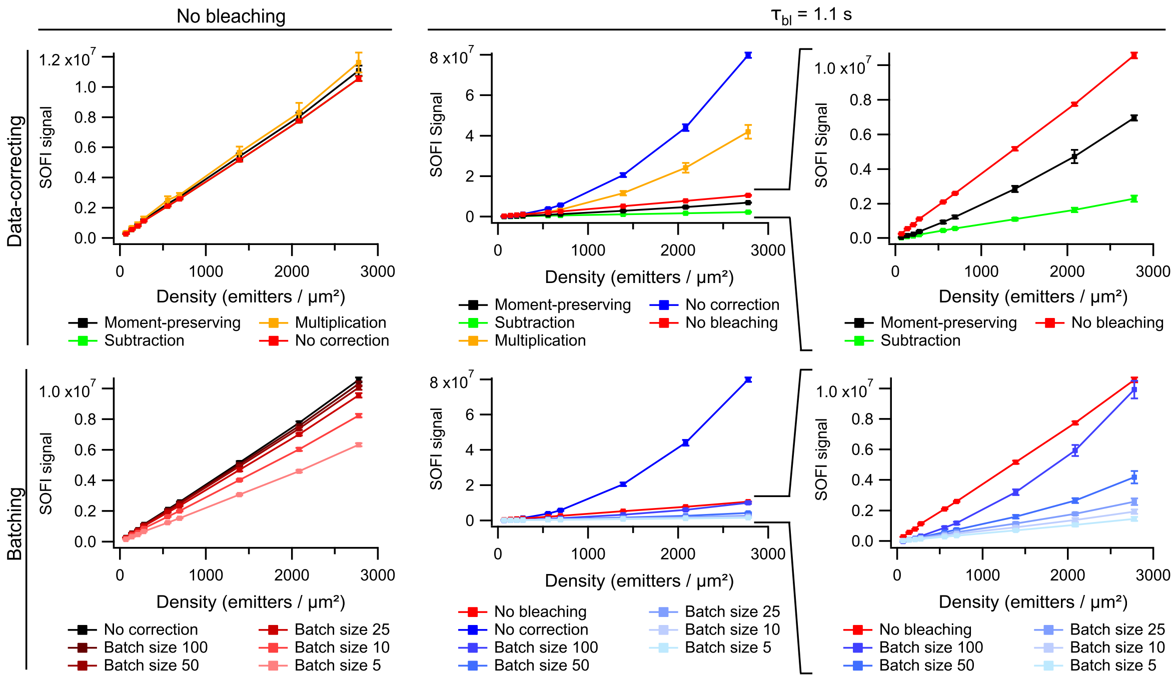

Figure S4: Analysis of photodestruction effects and correction methods on 2nd order SOFI signal with various emitter density. Each simulation contained 500 frames and was repeated 10-fold. Average SOFI signal is shown with standard deviation.

3 Correcting photodestruction in third order SOFI simulations

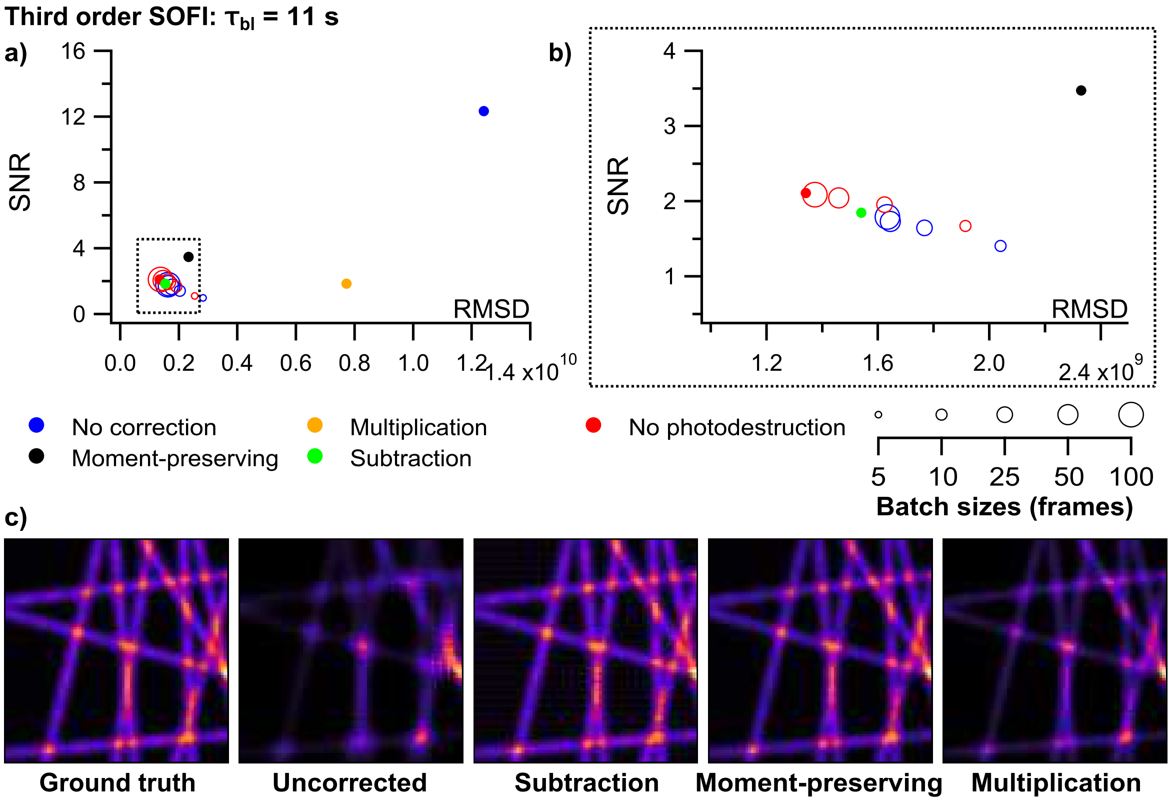

Figure S5: (a) Evaluation of different photodestruction-correcting methods on simulated data with fluorophores assigned a τ_{bl} value of 11 s for 3nd order SOFI calculations. Batch sizes of 100, 50, 25, 10, and 5 frames were examined and are visualized by different marker sizes. (b) Close-up of region in dotted box in (a). (c) Images are shown for certain datasets to serve as a visual reference for RMSD values. These images are averaged SOFI images of 100 repetitions.

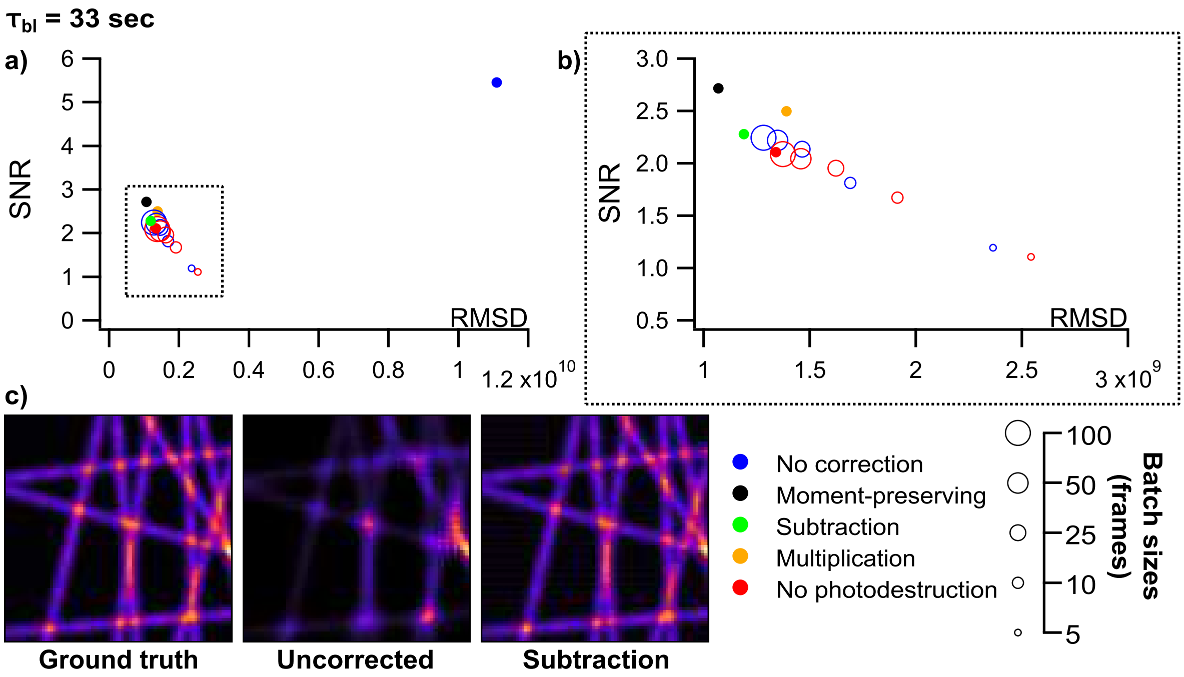

Figure S6: (a) Evaluation of different photodestruction-correcting methods on simulated data with fluorophores assigned a τ_{bl} value of 33 s for 3nd order SOFI calculations. Batch sizes of 100, 50, 25, 10, and 5 frames were examined and are visualized by different marker sizes. (b) Close-up of region in dotted box in (a). (c) Images are shown for certain datasets to serve as a visual reference for RMSD values. These images are averaged SOFI images of 100 repetitions. Deviations are attributed to insufficient convergence of the simulations.
